# Retracted: Optimum Rotation Speed for the Friction Stir Welding of Pure Copper

**DOI:** 10.1155/2014/285076

**Published:** 2014-10-29

**Authors:** International Scholarly Research Notices

This paper [1] has been retracted as it is essentially identical in content with the published article “Elucidating of rotation speed in friction stir welding of pure copper: Thermal modeling” published in Computational Materials Science, Volume 81, January 2014, Pages 296–302.
